# Health Tract, No. 90

**Published:** 1865

**Authors:** 


HEALTH TRACT, NO. 90

### SOLDIERS REMEMBERED

If you write to a soldier, friend, or relative in the army, using a common envelope and a sheet of foolscap-paper, you may also add, without exceeding the weight for which a three-cent postage-stamp will pay, as much tea as a teaspoon will take up twice, or as much black or cayenne pepper, such as is obtained from a good drug-store under the name of "Capsicum," as you can take up at once with a common teaspoon, and the smaller envelope of thin paper to hold either. Chewing the tea, a pinch at a time, every hour or half-hour, while keeping guard, or under circumstances of great thirst, or of excessive weariness or sleepiness, will enliven, will modify thirst, will invigorate, or will waken up to a grateful extent, considering the amount of tea used, and its perfect safety from ulterior ill results, such as follow the use of alcoholic drinks. But a heaping teaspoonful of genuine "Capsicum" is worth ten-fold its weight of tea-leaves, especially in summer, in many ways; for example, a single quarter of a pinch will save a man's life—that quarter of a pinch being put in a sleepy sentinel's eye. If it don't waken him up, and every body else within an Indian yell's distance, then it is not a prime article of capsicum. A single pinch in a glass of flat or warmish water will nullify these qualities, and besides satisfying thirst, will invigorate and effectually prevent that uncomfortable sensation arising from having drank largely of water. A good pinch, eaten at each meal, or whenever a cup of coffee or tea or water is swallowed, will always invigorate digestion, aids to prevent acidity, and is, besides, a great antagonist of the diarrhea, dysentery, flux, and "looseness," which are the great scourges of all armies. A level teaspoon of capsicum daily, taken in eating or drinking, or both, or if taken a pinch at a time during the day or night, would do more real good, and that without any ill result, than ten times the cost in rum and quinine, as a preventive against chills and fevers. Liquor and quinine initiate the soldier into intemperate habits; they will wake up a love, a craving, a slavery to strong drink, which pepper and water will never do. The latter invigorates like food, the former merely excites, then leaves weaker than before. A pinch of capsicum, which is simply pure cayenne pepper, will do a great deal more toward warming up a soldier, toward invigorating him, toward keeping him vigilant on guard, and toward modifying thirst or fatigue, than the best glass of grog ever swallowed. Capsicum goes farther, and is more efficient for all purposes, than black pepper; if by express or privately, send half a pound at a time, in a tin box. If you have nothing else to send in your letters, send a few pins, or a needle and some thread. Many have seen the time when a string or a pin would have been worth ten times its ordinary value. Write often to the soldier. Write long letters. Give all the news you can think of. Let every line be full of love; of kind, affectionate interest and encouragement. Be sure to inculcate a generous magnanimity toward those who oppose, *so as to have as few obstacles as possible to a cordial coming together again*, when that good time comes, as it certainly will, before long. We are all brethren, presently estranged, sons of the same sires, and, taking an enlarged view, the aggregate character is pretty well balanced

